# 

**Published:** 1887-10-22

**Authors:** 


CONTENTS.

**Medical Science at Perth** - - - - - - - 33
**Words of Consolation.**—Chapter LV. The Sufferings of Christ - - - - - - - - - 54
**Poetry:** Coming of Age - - - - - - - 54
**The Doctor's Pulpit.**—Cold Feet - - - - - 55
**Hospital Depression and the Registration of Nurses.** By Sir Andrew Clark, M.D., F.R.S. - - 56
**Oliver Wendell Holmes.** By Dr. Caterina - - - 57
**Amusements for Convalescents.**—Palmistry Notes. By a Lady - - - - - - - - 58
**At the Society of Arts on Wednesday.** By One who was There - - - - - - - - 59
**Notes and News.**—Miscellaneous Items.—Outbreak of Scarlet Fever at Liverpool.—Extension of the Royal South Hants Infirmary.—The Oswestry Cottage Hospital.—Hospital Saturday at Colchester.—Noble's Isle of Man Hospital.—The Outbreak of Small-pox at Sheffield.—Male Nurses for Hospital Patients.—Books and Newspapers for Hospitals.—Ladies as Dentists.—Kitchen Gardening at the Milton Fever Hospital.—Vacancies.—The New Circular Hospital at Hastings.—A Resignation.—Church Clergymen and Nonconformist Ministers.—The late Mr. W. C. Lysaght.—A Munificent Bequest.—Proposed Small-pox Hospital for Dundee.—Probationers and Kitchen Work.—The Montrose Asylum Board.—A Hospital Appointment, etc. - - - - - 60
**Annotations.**—"Granny."—Why Die of Fever?—"Brief Life" for the Doctor - - - - - - - - 63
**Amusements with Prizes, for all Ages.**—New Prize Competition.—For Men and Women.—The Children's Corner 64
**The National Pension Fund.** By Our Special Reporters 65
**Discussion on the Pension Fund Paper** - - 67
**East and West.** By Leslie Keith.—Chapter IV. - - 68

